# Characterizing drug allergy management among allergists in Canada: a national survey study

**DOI:** 10.1186/s13223-025-00981-4

**Published:** 2025-09-24

**Authors:** Erika Yue Lee, Brian Lee, Sinthiha Krishnan, Samira Jeimy, Matthieu Picard, Lana Rosenfield, Juan Ruiz, Christine Song

**Affiliations:** 1 Division of Allergy & Immunology, Department of Medicine, University of Toronto, Toronto, ON Canada; 2 Drug Allergy Clinic, Sunnybrook Health Sciences Centre, Toronto, ON Canada; 3 Division of Clinical Immunology and Allergy, Department of Medicine, Western University and Lawson Health Research Institute, London, ON Canada; 4 Faculty of Medicine, University of Toronto, Toronto, ON Canada; 5 Division of Allergy and Clinical Immunology, Hôpital Maisonneuve-Rosemont; Department of Medicine, Université de Montréal, Centre de Recherche de l’Hôpital Maisonneuve-Rosemont, Montreal, QC Canada; 6 Section of Allergy and Clinical Immunology, University of Manitoba, Winnipeg, MB Canada; 7 Division of Allergy & Immunology, University of British Columbia, Vancouver, British Columbia Canada; 8 Division of Allergy & Immunology, St. Michael’s Hospital, Toronto, ON Canada

**Keywords:** Drug allergy, Intradermal testing, Patch testing, Drug challenge

## Abstract

**Background:**

Unverified drug allergy labels are common and associated with significant patient harm, yet infrastructure and testing practices vary across clinical settings in Canada.

**Objective:**

To characterize variability in drug allergy management among allergists in Canada and identify setting-specific barriers to drug allergy testing and desensitization.

**Methods:**

We developed a peer-reviewed 40-item survey, distributed via the Canadian Society of Allergy and Clinical Immunology, to assess practice patterns, testing modalities, and perceived barriers among allergists. Descriptive statistics and Fisher’s exact test were used to evaluate responses by practice setting.

**Results:**

Sixty-six allergists responded (30% estimated response rate), with 48.4% solely practicing in community clinics and 21.9% solely in hospital-based clinics. While 87.9% performed some form of drug allergy testing, hospital-based allergists were significantly more likely to perform intradermal (81.1% vs. 48.7%, *p* = 0.004) and patch testing (38.2% vs. 8.8%, *p* = 0.009), as well as non-oral drug challenges (63.6% vs. 20.0%, *p* = 0.0005). Common barriers included a lack of nursing support and inadequate reimbursement.

**Conclusion:**

Drug allergy management practices vary substantially across Canada, with drug allergy testing being more frequently performed by allergists practicing in hospital-based clinics than by those in community-based clinics. Findings support the need for equitable access to testing infrastructure and system-level investments in improving drug allergy testing services.

**Supplementary Information:**

The online version contains supplementary material available at 10.1186/s13223-025-00981-4.

## Introduction

Drug allergy labels affect approximately 10–15% of patients in primary care, yet many are unverified and inaccurately persist in the medical record [1]. These labels can lead to suboptimal prescribing, increased risk of antimicrobial resistance, and increased healthcare costs [2, 3]. Therefore, accurate assessment of patients with suspected drug allergies provides benefit for the patients, their treating clinicians, and the healthcare system.

Allergists play a critical role in evaluating suspected drug allergies. After a thorough clinical history is taken about the initial drug allergic reaction, ancillary drug allergy testing is performed as indicated to guide management. Clinically available drug allergy testing tools in Canada include skin prick testing (SPT), intradermal testing (IDT), patch testing and drug challenges. The most recent Canadian Medical Association data suggest that only 25% of allergists practicing in Canada mainly work in academic health centres or hospitals whereas the remainder of allergists work in a community-based clinic [4]. Community-based allergists often lack the institutional resources found in hospitals, such as pharmacy, nursing staff, and emergency medical equipment, which serves as a barrier to performing adequate drug allergy testing for most practicing allergists.

There is limited data on drug allergy practice among allergists in Canada. Given that the majority of allergists practice outside of hospitals, understanding real-world capacity and constraints is critical to improving care access and outcomes. This study aims to characterize the current patterns of drug allergy assessment across Canada, evaluate variations in practice between hospital- and community-based settings, and explore perceived barriers to drug allergy testing. To our knowledge, this is the first national study to characterize drug allergy management practices among Canadian allergists, with direct comparisons across practice settings and an emphasis on access equity.

## Methods

The investigators, consisting of allergists practicing in various settings across Canada, developed an online survey that underwent iterative review for content and face validity. The final version was pilot-tested by five independent allergists and refined based on feedback prior to distribution. The survey consisted of 40 questions with close-ended questions to collect quantitative data and open-ended questions to collect qualitative data (Questionnaire available in Supplemental section). A response was not required for each question to complete the survey. The survey was disseminated to all eligible members of the Canadian Society of Allergy and Clinical Immunology (CSACI) as part of the monthly newsletter. All allergists actively practicing in Canada at the time of study were invited to participate in a voluntary manner and their responses remained anonymous. The survey was available for a total of 8 months from January to August of 2024.

Information regarding the demographics and characteristics of responders were collected, including their practice setting (type of practice, geographic area of practice). For the purpose of this study, a hospital-based clinic is defined as a clinic physically located in or attached to a hospital whereas a community-based clinic is defined as a clinic outside a hospital, regardless of the academic appointment of a practicing allergist. Questions addressed referral patterns for different drugs, the use of different methods of drug allergy skin testing (IDT or patch testing), the use of supervised drug challenges, drug desensitizations, as well as perceived barriers with any types of drug allergy testing. All data were captured anonymously using REDCap hosted at the University of Toronto. The study was approved by the institutional review ethics board.

Descriptive statistics were used to analyze responses for each item, including frequency and percentage, mean and standard deviation, or median and interquartile range depending on the type of data. Two-sided Fisher’s exact test was used to perform proportional comparisons at a prespecified α of 0.05 for significance. All statistical analyses were performed using SAS Studio.

## Results

### Characteristics of participants

The survey study included a total of 66 respondents. As of 2019, there were 219 registered Clinical Immunology & Allergy specialist physicians in Canada, which represents an estimated response rate of 30%.[4] Most respondents were based in Ontario (40.9%, *n* = 27) and Quebec (39.4%, *n* = 26). Almost half of them had been practicing for more than 10 years (47.4%, *n* = 31), compared to 23.1% (*n* = 15) practicing for 5 to 10 years and 29.2% (*n* = 19) practicing for fewer than 5 years. Among the 64 respondents, almost half practiced solely in the community setting (48.4%, *n* = 31), compared to 21.9% (*n* = 14) practiced solely in the academic setting and 29.7% (*n* = 19) had a mixed practice. About two-thirds of them saw both adults and children (67.2%, *n* = 43), compared to those who saw adults only (20.3%, *n* = 13) versus children only (12.5%, *n* = 8).

### Characteristics of drug allergy referral

Among the 64 respondents who answered the questions related to drug allergy referrals, almost all respondents saw at least one patient per week with drug allergy (96.9%, *n* = 62) but only 29.7% (*n* = 19) saw more than 10. Majority of the respondents reported that most drug allergy referrals came from family physicians (79.7%, *n* = 51), compared to 20.3% (*n* = 13) who reported most referrals came from emergency physicians, specialists, and others.

Among the 59 respondents who answered additional questions related drug allergy referrals, penicillin allergy was the most common drug allergy referral (86.4%, *n* = 51), followed by non-steroidal anti-inflammatory drug (NSAID) allergy (25.4%, *n* = 15) and cephalosporin allergy (22.0%, *n* = 13). Among the 58 respondents, 53.4% (*n* = 31) and 43.1% (*n* = 25) reported the average wait times for drug allergy referrals to be within 3 months and within 3 to 6 months, respectively, and 77.6% (*n* = 45) would perform drug allergy testing within 3 months.

Overall, majority of allergists performed drug allergy testing in their clinic (87.9%, *n* = 51/58), and more than half performed testing to any drugs (58.6%, *n* = 34/58). However, for drug allergy testing that was not readily available, 56.1% (*n* = 32/57) of respondents reported that they would refer the patient to another allergist who could do the testing and 35.1% (*n* = 20) reported that they would recommend to the patient to avoid culprit and cross-reactive drugs.

### Drug allergy testing

Among the 43 respondents who performed IDT as part of drug allergy assessment, 97.7% (*n* = 42) used IDT for immediate drug hypersensitivity reaction. Among the 30.3% (*n* = 20) of 66 respondents who performed patch testing, majority used for delayed drug hypersensitivity reactions (75.0%, *n* = 15). A summary of IDT and PT for the types of index reactions is shown in Table 1. Ex vivo testing was less commonly done; only 18.2% (*n* = 12/66) utilized them, with the majority being allergen-specific IgE (91.7%, *n* = 11).

**Table 1:** Types of drug allergy and skin testing

Types of drug allergy	IDT (65.2%, *n* = 43)	Patch testing (30.3%, *n* = 20)
Immediate	42 (97.7%)	1 (5%)
Delayed non-severe	22 (51.2%)	15 (75%)
SCAR AGEP SJS/TEN DRESS	17 (39.5%) 12 (66.7%) 3 (16.7%) 16 (88.9%)	15 (75%) 11 (73.3%) 6 (40.0%) 14 (93.3%)

Drug allergy testing differed significantly between community-based and hospital-based clinis. Except for oral challenge, allergists in the hospital setting performed more drug allergy skin testing and non-oral challenges compared to those in the community setting. * Subcutaneous, intramuscular or intravenous challenge; IDT, intradermal testing

**Fig. 1:**
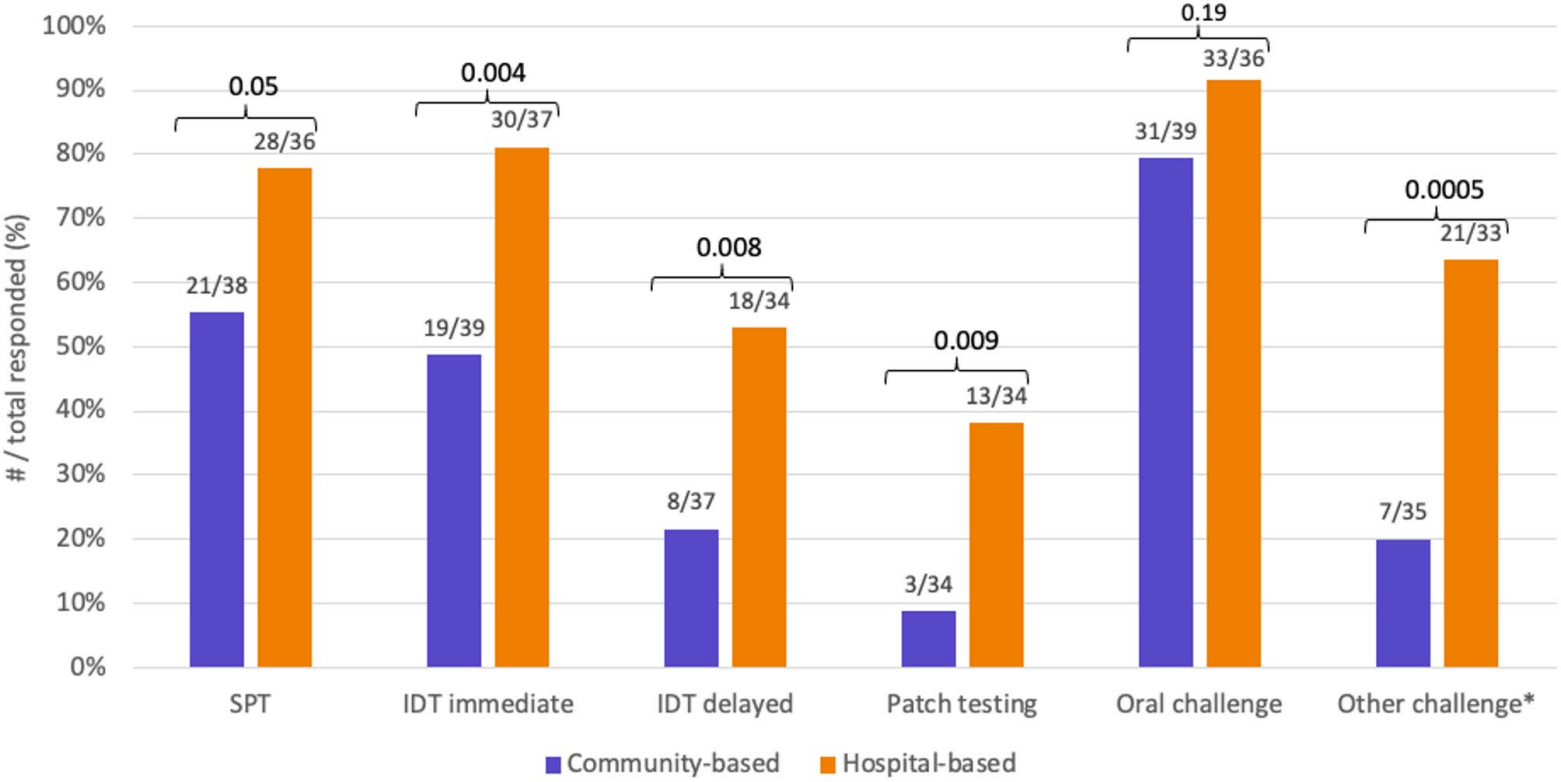
Comparison of drug allergy testing modalities by practice setting Drug allergy testing differed significantly between community-based and hospital-based clinis. Except for oral challenge, allergists in the hospital setting performed more drug allergy skin testing and non-oral challenges compared to those in the community setting. * Subcutaneous, intramuscular or intravenous challenge; IDT, intradermal testing

Compared to allergists with a community-based clinic, drug allergy skin testing (i.e. SPT, IDT and patch testing) was more often done by those with a hospital-based clinic Figure 1. Among the 49 respondents who performed observed drug challenges, similar pattern was seen for allergists with a hospital-based clinic versus community-based clinic Figure 1. Lastly, of 46 respondents, 63.0% (*n* = 29) performed drug desensitization for immediate and/or delayed drug hypersensitivity reactions whereas 37.0% (*n* = 17) did not perform this procedure.

Among the 42 respondents who provided information about drug allergy testing resources, 69.0% (*n* = 29) obtained drugs for allergy testing from a hospital pharmacy compared to 31.0% (*n* = 13) from a community pharmacy or 35.7% (*n* = 15) from other sources. Drug dilutions for allergy testing were often prepared by the clinic nursing staff (34.1%, *n* = 14/41), the hospital pharmacy (31.7%, *n* = 13/41), clinic physicians (24.4%, *n* = 10/41), and others (9.8%, *n* = 4/41).

### Barriers to drug allergy testing

**Fig. 2:**
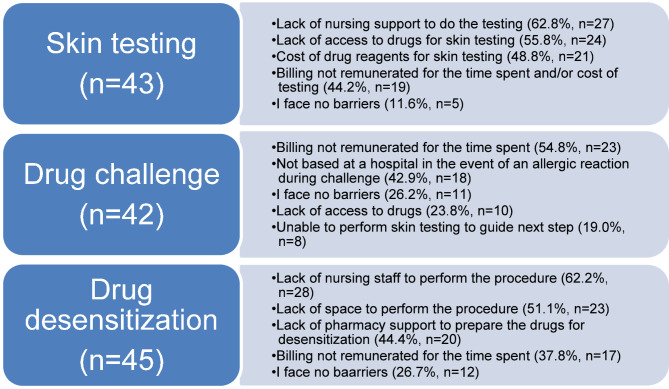
Barriers associated with drug allergy testing among allergists in Canada Lack of nursing support and inadequate remuneration were the two most commonly identified barriers by allergists regardless of their practice settings

We assessed possible barriers that allergists face for drug allergy testing. For skin testing, the most reported barrier is a lack of nursing support to perform the testing in 62.8% (*n* = 27) of the 43 respondents, compared to only 11.6% (*n* = 5) who reported no barriers. For drug challenge, the most reported barrier is billing not remunerated for the time spent and/or cost of testing in 54.8% (*n* = 23) of the 42 respondents, compared to 26.2% (*n* = 11) who reported no barriers. Lastly, for drug desensitization, the most reported barrier is a lack of adequate nursing staff to perform the procedure in 62.2% (*n* = 28) of the 45 respondents, compared to 26.7% (*n* = 12) with no barriers. Barriers associated with drug allergy testing are summarized in Fig. 2.

## Discussion

This is the first national survey to characterize drug allergy management practices among allergists in Canada. While referral for drug allergy was a significant part of practice for almost all respondents, advanced drug allergy testing that requires skin testing or parenteral drug challenges, was more frequently performed by allergists practicing in hospital-based clinics than by those in community-based clinics. The study identified potential barriers faced by allergists when conducting drug allergy testing. Despite the fact that drug allergy delabeling is critical for patient safety and antimicrobial stewardship, access to appropriate testing remains inconsistent across Canada. The findings underscore the need for equitable access to testing infrastructure and system-level investments in drug allergy services.

Antibiotic allergy, especially penicillin, was a common referral question. Family physicians were identified as a significant source of drug allergy referrals, speaking to the importance of primary care physicians in identifying patients needing accurate assessment for drug allergies. Family physicians are most likely to prescribe common antibiotics as well as aspirin and other NSAIDs for both cardiovascular and anti-inflammatory indications. Most of the allergists surveyed were able to see patients referred for drug allergy within 6 months of the referral being received. Depending on the type of referral, some patients can be tested on the first day of consultation, while others may need to return for testing. Most respondents reported being able to perform the testing within 3 months of consultation. While this may be a reasonable timeframe for non-urgent referrals, patients requiring more prompt assessment may be impacted. A future study looking at the impact of wait times for specific classes of medication allergies would better contextualize these numbers.

Although a large majority of allergists base their practice in the community setting, we found that drug allergy testing is mostly done in the hospital setting. While the majority of respondents perform skin testing (likely drug-specific) and observed challenges in their practices, over half of them still need to refer patients to another allergist, likely the one with hospital-based clinic, for certain medication allergies. Major barriers in performing drug allergy assessment were lack of nursing and pharmacy support, difficulty in obtaining medications for skin testing, concerns around managing severe reactions, and low remuneration relative to cost likely result in most drug allergy testing being done in a hospital setting. At present, our survey showed that almost half of the allergists in Canada do not have hospital-based clinics that would allow them to conduct complex drug allergy testing. One main reason is that the majority of hospitals in Canada do not have an allergy and immunology clinic on site. The disproportionate availability of hospital-based allergy services (despite the decentralized distribution of allergists) raises important concerns about equitable access to drug allergy evaluation across the Canadian healthcare system.

Further, our survey showed that most of the drugs for allergy testing were obtained from hospital-based pharmacies. Allergists most often depend on their nursing staff or the hospital-based pharmacy to prepare dilutions for skin testing. The associated costs of medications and pharmacy resources are substantial and represent a significant barrier. Allergists have to cover the cost of pharmacy resources and purchase the drugs out-of-pocket in many cases either directly or indirectly through their hospital or community clinic overhead. However, numerous studies have shown the cost-effectiveness of drug allergy testing, especially for antibiotics, in addition to improving patient outcomes and reducing the risk of antimicrobial resistance [3, 5–8]. As such, these operational factors are crucial when considering scale-up efforts or the development of regional referral hubs for drug allergy testing.

Systemic drug challenges are considered the gold standard in drug allergy assessment. Oral drug challenge was the most common type of testing performed by survey respondents, presumably due to a combination of the ease of administration and the most common drugs referred for allergy testing being most often administered orally. For patients with low-risk index drug reactions, an observed oral challenge can be considered without skin testing for certain medications. However, for many moderate-to-severe reactions, skin testing is advised before considering an oral challenge. Our study showed that more complex drug allergy testing including parenteral drug challenges is more often done by allergists in hospital-based clinics, highlighting the need to increase allergy presence in the hospital setting to provide advanced drug allergy care.

Comprehensive drug allergy testing enables the removal of inaccurate or unverified drug allergy labels, which benefits patients, clinicians, hospitals, and the Canadian healthcare system. Appropriate assessment and confirmation of true drug allergies also help prevent adverse outcomes and allows for the utilization of drug desensitization for life-saving treatments. The study highlights the importance of hospital-based allergy clinics in providing complex drug allergy assessment and desensitization. As hospitals examine their strategic priorities in ambulatory care, the study underscores the need for collaboration between pharmacy, nursing, and physician teams to advocate for the resources required to provide and expand this essential care for patients.

A key strength of this study is its national scope and high estimated response rate (30%) relative to the total number of practicing allergists in Canada. Limitations included missing data as respondents did not have to answer every question and self-reporting of data. For some testing modalities such as patch testing and parenteral challenge, small sample sizes limit the generalizability of findings. These data should be interpreted as indicative rather than definitive. Additionnaly, allergists who routinely perform drug allergy testing may have been more likely to complete the survey compared to those who do not. As a result, drug allergy evaluations may be less commonly performed among Canadian allergists than the survey suggests, and some barriers may not have been captured. Lastly, while our sample included responses from across Canada, the geographic distribution skewed heavily toward Ontario and Quebec. This reflects known workforce clustering in these provinces and is consistent with CSACI membership patterns. Nonetheless, perspectives from underrepresented provinces merit further targeted study.

## Conclusion

To our knowledge, this is the first survey that characterized the practice of drug allergy management among allergists in Canada. Practice patterns may vary depending on the clinical setting, including geographic location, whether the allergist is hospital- or community-based, and resource limitations; advanced drug allergy testing was more often conducted by allergists practicing in a hospital setting. These findings suggest potential benefit in supporting hospital-based allergy services and creating shared testing infrastructure to address gaps in comprehensive drug allergy care across Canada.

## Supplementary Information

Below is the link to the electronic supplementary material.

Supplementary Material 1

## Data Availability

No datasets were generated or analysed during the current study.

## Abbreviations

SPT: Skin prick testing
IDT: Intradermal testing
CSACI: Canadian Society of Allergy and Clinical Immunology
NSAID: Nonsteroidal anti-inflammatory drug
SCAR: Severe cutaneous adverse reaction
AGEP: Acute generalized exanthematous pustulosis
SJS: Stevens-Johnson syndrome
TEN: Toxic epidermal necrolysis
DRESS: Drug reaction with eosinophilia and systemic symptoms

## References

[1] Singer AG, Kosowan L, Nankissoor N, Phung R, Protudjer JLP, Abrams EM. Use of electronic medical records to describe the prevalence of allergic diseases in Canada. Allergy Asthma Clin Immunol. 2021;17(1):85. 34407859 10.1186/s13223-021-00580-z PMC8371898

[2] Blumenthal KG, Shenoy ES, Huang M, Kuhlen JL, Ware WA, Parker RA et al. Socio GV, editor. PLoS One. The Impact of Reporting a Prior Penicillin Allergy on the Treatment of Methicillin-Sensitive Staphylococcus aureus Bacteremia. De 2016; 11(7):e0159406. Available from: https://journals.plos.org/plosone/article?id=10.1371/journal.pone.0159406 10.1371/journal.pone.0159406 PMC4954694 27438379

[3] Macy E, Contreras R. Health care use and serious infection prevalence associated with penicillin allergy in hospitalized patients: A cohort study. J Allergy Clin Immunol. 2014;133(3):790–6. 24188976 10.1016/j.jaci.2013.09.021

[4] Canadian Medical Association. Number of Physicians by Province/Territory and Specialty, Canada, 2019. Canadian Medical Association 2019 [cited 2025 Feb 22]. Available from: https://www.cma.ca/canadian-physician-demographics-and-supply-archive

[5] Huang KG, Cluzet V, Hamilton K, Fadugba O. The Impact of Reported Beta-Lactam Allergy in Hospitalized Patients With Hematologic Malignancies Requiring Antibiotics [published correction appears in Clin Infect Dis. 2018;67(7):1151]. Clin Infect Dis. 2018;67(1):27–33. 10.1093/cid/ciy037 29346543

[6] Wu JH, Langford BJ, Schwartz KL, et al. Potential negative effects of antimicrobial allergy labelling on patient care: A systematic review. Can J Hosp Pharm. 2018;71(1):29–35. 29531395 PMC5842048

[7] Picard M, Begin P, Bouchard H, Cloutier J, Lacombe-Barrios J, Paradis J, et al. Treatment of patients with a history of penicillin allergy in a large tertiary-care academic hospital. J Allergy Clin Immunol Pract. 2013;1:252–7. 24565481 10.1016/j.jaip.2013.01.006

[8] Macy E, Shu YH. The effect of penicillin allergy testing on future health care utilization: A matched cohort study. J Allergy Clin Immunol Pract. 2017;5(3):705–10. 28366717 10.1016/j.jaip.2017.02.012

