# Supplementary material for: Characterizing drug allergy management among allergists in Canada: a national survey study
Source: Allergy Asthma Clin Immunol. 2025 Sep 24;21:41. doi: 10.1186/s13223-025-00981-4 (PMC12462252; doi:10.1186/s13223-025-00981-4)
Supplement: Supplementary file 1 — Supplementary Material 1 [file 13223_2025_981_MOESM1_ESM.pdf]

# The DAM-C Study: A Survey Study on Drug Allergy Management in Canada

Dear Colleagues:

We are inviting you to participate in an online survey that aims to characterize the current practice of drug allergy management in Canada. The survey is being conducted through REDCap provided by the University of Toronto and will take about 10 minutes or less to complete. Your participation is voluntary, and your responses will remain anonymous.

There are no anticipated benefits or risks to complete this survey. However, future research may benefit from the knowledge gained from this survey. By completing this survey, you provide us the consent for your participation.

If you have any questions, please email Erika Lee (erika.lee@mail.utoronto.ca).

Thank you for your participation.

The DAM-C Study Group

---

1 Which province or territory do you practice allergy & immunology in Canada?

- ◯ Alberta
- ◯ British Columbia
- ◯ Manitoba
- ◯ New Brunswick
- ◯ Newfoundland and Labrador
- ◯ Nova Scotia
- ◯ Ontario
- ◯ Prince Edward Island
- ◯ Quebec
- ◯ Saskatchewan
- ◯ Northwest Territories
- ◯ Nunavut
- ◯ Yukon

---

2 Which population of patients do you see?

- ◯ Children only
- ◯ Adults only
- ◯ Both - indicate __% children and __% adults ______

---

3 What is your practice setting?

- ◯ Academia
- ◯ Community
- ◯ Both - indicate __% academia and __% community ______

---

4 Where do you practice?

- ◯ Hospital-based clinic
- ◯ Community-based clinic
- ◯ Both - indicate __% hospital and __% community ______

---

5 How long have you been practicing in the field of allergy & immunology?

- ◯ < 5 years
- ◯ 5 to 10 years
- ◯ > 10 years

---

6 What is your assigned sex at birth?

- ◯ Female
- ◯ Male
- ◯ Prefer not to answer
- ◯ Other ______

| | | |
|---|---|---|
| 7 | Which age group best describes you? | ◯ < 30 years old<br>◯ 30 to 39 years old<br>◯ 40 to 49 years old<br>◯ 50 to 59 years old<br>◯ 60 years old and above |
| 8 | What best describes your training before allergy & immunology? | ◯ Pediatrics<br>◯ Internal Medicine<br>◯ Others, please specify<br>__________ |
| 9 | How many patients do you see in your allergy & immunology clinic on average per week? | ◯ < 50 patients per week<br>◯ 50 to 100 patients per week<br>◯ > 100 patients per week |

## Drug Allergy Management

| | | None | 1 to 5 patients per week | 5 to 10 patients per week | 11 to 15 patients per week | 16 to 20 patients per week | > 20 patients per week |
|---|---|---|---|---|---|---|---|
| 10 | How many drug allergy consultations do you see on average per week? | ◯ | ◯ | ◯ | ◯ | ◯ | ◯ |

11 Where do most of your drug allergy referrals come from?

- ◯ Family physicians
- ◯ Emergency physicians
- ◯ Specialists, please specify ______
- ◯ Others, please specify ______

## 12) How often do you see the following drug allergy referrals in your clinic?

| | frequently (at least once per week) | sometimes (at least once per month) | occasionally (at least once per year) | none |
|---|---|---|---|---|
| Penicillins | ◯ | ◯ | ◯ | ◯ |
| Carbapenems | ◯ | ◯ | ◯ | ◯ |
| Cephalosporins | ◯ | ◯ | ◯ | ◯ |
| Clindamycin | ◯ | ◯ | ◯ | ◯ |
| Macrolides | ◯ | ◯ | ◯ | ◯ |
| Metronidazole | ◯ | ◯ | ◯ | ◯ |
| Quinolones | ◯ | ◯ | ◯ | ◯ |
| Sulfonamides | ◯ | ◯ | ◯ | ◯ |
| Vancomycin | ◯ | ◯ | ◯ | ◯ |
| Local anesthetics | ◯ | ◯ | ◯ | ◯ |
| General anesthetics | ◯ | ◯ | ◯ | ◯ |
| NSAIDs | ◯ | ◯ | ◯ | ◯ |
| Acetaminophen | ◯ | ◯ | ◯ | ◯ |
| Opiates | ◯ | ◯ | ◯ | ◯ |
| Iodine-based radiocontrast | ◯ | ◯ | ◯ | ◯ |
| Gadolinium-based radiocontrast | ◯ | ◯ | ◯ | ◯ |
| Monoclonal antibodies | ◯ | ◯ | ◯ | ◯ |
| Chemotherapeutic agents | ◯ | ◯ | ◯ | ◯ |
| Intravenous iron | ◯ | ◯ | ◯ | ◯ |
| Other drugs (Specify name and frequency based on the scale above). ______ | ◯ | ◯ | ◯ | ◯ |

13 What is the average wait time in your clinic for drug allergy referrals?

- ◯ < 1 month
- ◯ 1 to 3 months
- ◯ 3 to 6 months
- ◯ > 6 months
- ◯ Other, please specify ______

14 Do you perform any drug allergy testing (i.e. skin prick test, intradermal test, patch test, or drug challenge) in your clinic?

- ◯ No
- ◯ Yes, penicillin only
- ◯ Yes, only certain drugs (please list the drugs) ______
- ◯ Yes, whenever indicated
- ◯ Other ______

15 For the drug(s) that you are not able to perform drug allergy testing, what do you usually recommend? (Check all that apply)

- ☐ Refer to another allergist if I think skin testing or challenge is available and helpful
- ☐ Avoid the culprit drug(s) and potentially cross-reactive ones
- ☐ Other, please specify. ______

## Drawing allergy skin testing

| | | < 1 month (Specify drugs) ______ | 1 to 3 months | 3 to 6 months | > 6 months |
|---|---|---|---|---|---|
| 16 | When do you usually perform drug skin allergy testing after initial consultation? | ◯ | ◯ | ◯ | ◯ |

**17) If you practice in a community-based clinic, which of the following drug allergy testing do you perform? (Please skip if you practice in a hospital-based clinic only)**

| | Yes | No |
|---|---|---|
| Skin prick testing | ◯ | ◯ |
| Intradermal testing with immediate reading | ◯ | ◯ |
| Intradermal testing with delayed reading | ◯ | ◯ |
| Patch testing | ◯ | ◯ |
| Oral drug challenge | ◯ | ◯ |
| Intramuscular drug challenge | ◯ | ◯ |
| Intravenous drug challenge | ◯ | ◯ |
| Other, please specify ______ | ◯ | ◯ |

**18) If you practice in a hospital-based clinic, which of the following drug allergy testing do you perform? (Please skip if you practice in a community-based clinic only)**

| | Yes | No |
|---|---|---|
| Skin prick testing | ◯ | ◯ |
| Intradermal testing with immediate reading | ◯ | ◯ |
| Intradermal testing with delayed reading | ◯ | ◯ |
| Patch testing | ◯ | ◯ |
| Oral drug challenge | ◯ | ◯ |
| Subcutaneous drug challenge | ◯ | ◯ |
| Intramuscular drug challenge | ◯ | ◯ |
| Intravenous drug challenge | ◯ | ◯ |
| Other, please specify. ______ | ◯ | ◯ |

**19) How often do you perform intradermal testing for the following drug classes?**

| | frequently (at least once per week) | sometimes (at least once per month) | occasionally (at least once per year) | none |
|---|---|---|---|---|
| Penicillins | ○ | ○ | ○ | ○ |
| Carbapenems | ○ | ○ | ○ | ○ |
| Cephalosporins | ○ | ○ | ○ | ○ |
| Clindamycin | ○ | ○ | ○ | ○ |
| Macrolides | ○ | ○ | ○ | ○ |
| Metronidazole | ○ | ○ | ○ | ○ |
| Quinolones | ○ | ○ | ○ | ○ |
| Sulfonamides | ○ | ○ | ○ | ○ |
| Vancomycin | ○ | ○ | ○ | ○ |
| Local anesthetics | ○ | ○ | ○ | ○ |
| General anesthetics | ○ | ○ | ○ | ○ |
| NSAIDs | ○ | ○ | ○ | ○ |
| Opiates | ○ | ○ | ○ | ○ |
| Iodine-based radiocontrast | ○ | ○ | ○ | ○ |
| Gadolinium-based radiocontrast | ○ | ○ | ○ | ○ |
| Monoclonal antibodies | ○ | ○ | ○ | ○ |
| Chemotherapeutic agents | ○ | ○ | ○ | ○ |
| Other drugs (Specify) ______ | ○ | ○ | ○ | ○ |

20 Which types of index drug reactions do you perform intradermal testing to culprit drugs? (Check all that apply)

- ☐ Immediate type 1 drug reactions
- ☐ Delayed non-severe type 4 drug reactions
- ☐ Severe cutaneous adverse drug reactions
- ☐ Others

______

21 Which types of suspected or confirmed severe cutaneous adverse reactions have you performed intradermal testing to culprit drugs? (Check all that apply)

- ☐ Acute Generalized Exanthematous Pustulosis (AGEP)
- ☐ Stevens-Johnson Syndrome / Toxic Epidermal Necrolysis (SJS/TEN)
- ☐ Drug Reaction with Eosinophilia and Systemic Symptoms (DRESS)
- ☐ Others (specify) ______

22 Which types of index drug reactions do you perform patch testing to culprit drugs? (Check all that apply)

- ☐ Immediate type 1 drug reactions
- ☐ Delayed non-severe type 4 drug reactions
- ☐ Severe cutaneous adverse drug reactions
- ☐ Others ______

23 Which types of suspected or confirmed severe cutaneous adverse reactions have you performed patch testing to culprit drugs? (Check all that apply)

- ☐ Acute Generalized Exanthematous Pustulosis (AGEP)
- ☐ Stevens-Johnson Syndrome / Toxic Epidermal Necrolysis (SJS/TEN)
- ☐ Drug Reaction with Eosinophilia and Systemic Symptoms (DRESS)
- ☐ Others (specify) ______

## 24) How often do you perform patch testing for the following drug classes?

| | frequently (at least once per week) | sometimes (at least once per month) | occasionally (at least once per year) | none |
|---|---|---|---|---|
| Penicillins | ◯ | ◯ | ◯ | ◯ |
| Carbapenems | ◯ | ◯ | ◯ | ◯ |
| Cephalosporins | ◯ | ◯ | ◯ | ◯ |
| Clindamycin | ◯ | ◯ | ◯ | ◯ |
| Macrolides | ◯ | ◯ | ◯ | ◯ |
| Metronidazole | ◯ | ◯ | ◯ | ◯ |
| Quinolones | ◯ | ◯ | ◯ | ◯ |
| Sulfonamides | ◯ | ◯ | ◯ | ◯ |
| Vancomycin | ◯ | ◯ | ◯ | ◯ |
| Local anesthetics | ◯ | ◯ | ◯ | ◯ |
| General anesthetics | ◯ | ◯ | ◯ | ◯ |
| NSAIDs | ◯ | ◯ | ◯ | ◯ |
| Opiates | ◯ | ◯ | ◯ | ◯ |
| Iodine-based radiocontrast | ◯ | ◯ | ◯ | ◯ |
| Gadolinium-based radiocontrast | ◯ | ◯ | ◯ | ◯ |
| Monoclonal antibodies | ◯ | ◯ | ◯ | ◯ |
| Chemotherapeutic agents | ◯ | ◯ | ◯ | ◯ |
| Other drugs (Specify) ______ | ◯ | ◯ | ◯ | ◯ |

25 If you perform skin testing, where do you find drug concentrations for skin allergy testing? (Check all that apply)

- ☐ AAAAI Drug Allergy Practice Parameters
- ☐ EAACI Position Papers
- ☐ Published peer-reviewed papers
- ☐ From experienced colleagues
- ☐ From personal experience
- ☐ CSACI drug allergy position statement
- ☐ Others ______

26 Where do you obtain drugs for skin allergy testing? (Check all that apply)

- ☐ Hospital pharmacy
- ☐ Community pharmacy
- ☐ Pharmaceutical company (e.g. Pre-Pen from ALK)
- ☐ Others (Specify) ______

27 Who prepares most of the drug dilutions for skin allergy testing?

- ◯ Hospital-based pharmacy
- ◯ Community compounding pharmacy
- ◯ Clinic nurses
- ◯ Clinic physicians
- ◯ Others (Specify) ______

28 Who pays for the drugs used for skin allergy testing?

- ◯ Hospital
- ◯ Office
- ◯ Allergists
- ◯ Others ______

29 Do you send your patients with drug allergy for ex vivo drug testing?

◯ Yes ◯ No

30 Which of the following ex-vivo drug testing have you sent? (Click all that apply)

- ☐ Lymphocyte transformation test (LTT)
- ☐ Basophil activation test (BAT)
- ☐ Enzyme-linked immunoSpot (ELISpot)
- ☐ Allergen-specific IgE levels
- ☐ Others ______

## Drug challenge

31 Do you perform observed drug challenge (also known as drug provocation testing) in your clinic?

◯ Yes ◯ No

32 Do you perform skin testing before drug challenge whenever available?

◯ Never
◯ Sometimes (< 50%)
◯ Most of the time (>50%)
◯ Always

33 Where do you perform the observed drug challenge? (Check all that apply)

☐ In a hospital setting
☐ In a community office next to a hospital
☐ In a community office not attached to a hospital
☐ Others ______

**34) How often do you perform the following types of drug challenges?**

| | frequently (at least once per week) | sometimes (at least once per month) | occasionally (at least once per year) | none |
|---|---|---|---|---|
| Oral | ◯ | ◯ | ◯ | ◯ |
| Subcutaneous | ◯ | ◯ | ◯ | ◯ |
| Intravenous | ◯ | ◯ | ◯ | ◯ |
| Intramuscular | ◯ | ◯ | ◯ | ◯ |
| Other ______ | ◯ | ◯ | ◯ | ◯ |

**35) Do you feel comfortable for direct observed drug challenge without prior skin testing for the following drugs when the history is considered low risk?**

| | Yes | No |
|---|---|---|
| Penicillins | ◯ | ◯ |
| Carbapenems | ◯ | ◯ |
| Cephalosporins | ◯ | ◯ |
| Clindamycin | ◯ | ◯ |
| Macrolides | ◯ | ◯ |
| Metronidazole | ◯ | ◯ |
| Quinolones | ◯ | ◯ |
| Sulfonamides | ◯ | ◯ |
| Vancomycin | ◯ | ◯ |
| Local anesthetics | ◯ | ◯ |
| NSAIDs | ◯ | ◯ |
| Opiates | ◯ | ◯ |
| Iodine-based radiocontrast | ◯ | ◯ |
| Gadolinium-based radiocontrast | ◯ | ◯ |
| Monoclonal antibodies | ◯ | ◯ |
| Chemotherapeutic agents | ◯ | ◯ |

36 Do you perform drug desensitization when medically indicated?

- ◯ Yes, for immediate drug hypersensitivity reactions only
- ◯ Yes, for both immediate and delayed drug hypersensitivity reactions
- ◯ No

37 What are some of the barriers you face to performing drug desensitization?

- ☐ Lack of space to perform the procedure
- ☐ Lack of nursing staff to perform the procedure
- ☐ Lack of pharmacy support to prepare the drugs for desensitization
- ☐ Billing not renumerated for the time spent
- ☐ I face no barriers
- ☐ Others (specify) ______

38 Where do you perform drug desensitization procedures?

- ◯ Day unit
- ◯ Inpatient ward
- ◯ ICU
- ◯ Others ______

## Barriers to Drug Allergy Testing

| | | Lack of access to drugs for skin testing | Cost of drug reagents for skin testing | Lack of nursing support to do the testing | Billing not renumerated for the time spent and/or cost of testing | I face no barriers | Other, please specify ______ |
|---|---|---|---|---|---|---|---|
| 39 | What are some barriers to conducting drug allergy skin testing in your office? (Check all that apply) | ☐ | ☐ | ☐ | ☐ | ☐ | ☐ |

40 What are some barriers to conducting observed drug challenge in your office? (Check all that apply)

- ☐ Unable to perform drug allergy skin testing to guide next step
- ☐ Lack of access to drugs to conduct the drug challenge
- ☐ Not based at a hospital in the event of an allergic reaction during challenge
- ☐ Billing not renumerated for the time spent on the procedure
- ☐ I face no barriers
- ☐ Others (please specify) ______

Do you have any general comment or feedback?

__________________________________________
